# Increasing Patient Safety Event Reporting Among Pediatric Residents

**DOI:** 10.7759/cureus.23298

**Published:** 2022-03-18

**Authors:** Vini Vijayan, Jolie Limon

**Affiliations:** 1 Pediatrics, Valley Children’s Healthcare, Madera, USA

**Keywords:** pediatrics, graduate medical education, resident physicians, patient safety, patient safety events

## Abstract

Background and objective

Despite their role as frontline providers, resident physicians often underreport adverse patient events or safety issues they encounter. The objective of this study was to increase the number of patient safety events (PSE) reported at our institution through the implementation of a longitudinal, multi-pronged approach.

Methods

We designed and implemented a series of interventions focused on increasing patient safety events reported by resident physicians from October 2018 to April 2021. Interventions consisted of formal didactic sessions, increasing awareness among organizational leaders about the integral role of residents, implementing a direct feedback process to residents regarding the events, and encouraging them to develop solutions to their PSE that were associated with a financial incentive. We collected the rates of reports every month to assess the impact of our interventions.

Results

The mean number of PSEs submitted monthly increased from zero to two reports at baseline to 10.4 during the study period. The mean number of PSE increased to 5.8 (range: 2-11) at the end of the first intervention. Following the third intervention, the average number of reported PSE was 12.3 (range: 5-18). There was a continued increase in the number of events reported across the study period, which was sustained. The outcome of interest was not achieved after intervention 1 but was achieved in 27% and 62% of months following interventions two and three. By theend of the study period, our goal of >13 PSEs per month was consistently met. The most significant increase in reporting occurred when residents received positive timely feedback regarding their reports.

Conclusions

The number of patient safety events reported by pediatric residents increased at our institution following the implementation of a multi-pronged approach including enhanced education, recognition of the residents as frontline reporters among institutional stakeholders, and direct feedback regarding submissions. Our strategies may be replicated at other residency programs seeking to establish resident involvement in safety initiatives. Further work is necessary to ensure residents gain an understanding of how patient safety events are addressed and prevented at an organizational level.

## Introduction

The Accreditation Council for Graduate Medical Education (ACGME) requires that graduate medical education programs ensure resident engagement in quality improvement and patient safety activities and promote a culture of safety [1]. Since the implementation of the Clinical Learning Environment Review (CLER) patient safety pathway in 2016, patient safety and quality improvement initiatives have been recognized as paramount in the training of new physicians [2,3].

As frontline providers, resident physicians are often the first to encounter and learn of an adverse patient event or safety issue. However, several studies have demonstrated the under-reporting of patient safety events (PSE) among physicians, particularly among residents and medical students [3-6]. Hatoun et al. identified barriers to safety reporting, including lack of time, tedious reporting processes, fear of retaliation or punishment, lack of feedback regarding action taken following submission, and the perception of residents as temporary providers [6]. An additional obstacle to error reporting included the inability to determine what comprised a safety event [7,8].

Previous studies directed towards residents have utilized various educational interventions and financial incentives to increase reporting [8-12]. However, despite efforts to promote the integration of trainees in the organizational patient safety and quality infrastructure, reporting of PSE by residents remains low [13]. A literature review by Kaldjian et al. identified specific motivators that encouraged reporting medical errors [14]. These included feelings of personal and professional integrity, a sense of personal responsibility to share lessons learned from mistakes, and the potential for error reporting to improve the care of future patients [14,15].

We began tracking our resident PSE submissions in July 2017. Like other graduate medical education programs, we found that only a few of our trainees submitted reports [15,16]. The baseline number of PSE reported by resident physicians ranged from zero to two reports per month.

The objective of this study was to increase the number of patient safety events (PSEs) reported at our institution from 0.6 PSE reports per month to >13 reports per month through the implementation of a longitudinal, multi-pronged approach from October 2018 to April 2021. We developed various interventions that were in alliance with our sponsoring institution’s patient safety and quality improvement mission. This included increased safety events and error reporting by residents and integration of residents into the organizational safety culture.

## Materials and methods

This study was conducted at Valley Children’s Hospital (VCH), a 330-bed free-standing children’s hospital located in Madera, California, from October 2018 to April 2021. The participants included resident physicians at the VCH pediatric residency training program. The program is accredited by the Accreditation Council for Graduate Medical Education (ACGME) and has 13 residency positions per post-graduate year for a total of 39 positions. This study was deemed exempt by the institutional review board.

We designed a series of interventions focused on increasing patient safety events reported by resident physicians. These consisted of education for residents regarding patient safety, increased awareness of the CLER requirements among organizational leaders, a direct feedback process to residents regarding events, and encouraging residents to develop hypothetical solutions to patient safety events to further increase resident engagement. Each phase of the study was designed to address barriers reported in the literature and encourage and empower residents to become a part of the safety culture at our institution. Our interventions were initiated in October 2018 and reinforced over the study period of 30 months with completion of data gathering in April 2021. We monitored and recorded the rates of reporting each month and calculated the average reporting rates after each phase to assess the impact of the intervention.

Patient safety event reporting system 

At our institution, patient safety events are reported through an electronic reporting system, MorCare^TM^ (Chicago, IL: MorCare LLC). This system was adopted in 2017 to streamline the reporting process. Hospital employees can access the system through the hospital intranet system, and reports are not linked to the electronic medical record (EMR). MorCare^TM^ is available for use in the inpatient and outpatient settings.

The system allows employees to report information that includes person(s) involved in the event, a description of the event, and the reporter’s title. Reporters also can submit reports anonymously. There is a drop-down box to identify whether the reporter is a resident physician. The system routes all PSEs to the institutional patient safety and quality team, the chief nursing officer, and the chief medical officer for review daily. PSEs submitted by a resident or about a resident are referred to the Designated Institutional Official (DIO), Program Director, and graduate medical education (GME) manager for review. Any immediate safety needs specific to residents are addressed immediately.

At baseline, education regarding patient safety is provided to all residents and includes expectations for reporting and mechanisms for reporting a patient safety event (PSE). These training sessions are mandatory and are completed during intern orientation and then yearly for all residents.

Safety events and definitions

As per the National Safety Forum definition, safety events included serious reportable events and those incidents that met our institution’s standards for reporting, such as near misses and unsafe conditions. We defined the number of reported patient safety events (PSEs) as the number of events reported per month by resident physicians. We recorded resident-reported PSEs in our internal database to monitor and assess trends in reporting.

Interventions

The first intervention (October 2018 to March 2019) focused on providing education for residents regarding patient safety and error reporting. Residents received education regarding patient safety as a series of didactic lectures to supplement the training provided during orientation and organizational leadership about the need for resident involvement in our current systems. The lectures were delivered during a protected lecture period and highlighted the impact of patient safety event reporting to detect latent safety issues. The lectures also provided examples of well-written PSE reports, shared information about the CLER pathways, and provided demonstrations of the electronic reporting system. In addition, the sessions emphasized the integral role of residents as frontline witnesses to safety events, their ability to mitigate lapses in safety, and serve as safety champions. We also reinforced that reporting was not punitive and encouraged residents to submit at least four PSEs per year. The sessions were delivered beginning in October 2018 and repeated multiple times during the course of the other interventions to capture all residents.

The second phase of our intervention (April 2019 to February 2020) focused on efforts to build a consensus among organizational leaders and stakeholders to support resident involvement in the organization's safety culture and cultivate an awareness of the CLER requirements among this group. The Designated Institutional Official (DIO) met with senior leadership and educated them on the ACGME and CLER requirements regarding resident involvement in organizational safety initiatives. As a result, the metric of “resident generated PSEs” was added to the organizational scorecard. The resident-generated PSE metric is presented to the board of trustees monthly by the chief executive officer. This scorecard which also includes other key safety and quality metrics (e.g. central line bloodstream infections, unplanned extubations, ventilator-associated pneumonia, and readmission rates) is displayed on our intranet site for hospital employees and residents to view. This intervention highlighted the value of residents and residents’ involvement and commitment to cultivating a culture of safety at our institution.

The third phase of our intervention (March 2020 to October 2020) involved creating a feedback process regarding the residents’ PSE submissions, thereby building an environment that allowed for open communication about unsafe situations and disseminate system-level changes that occurred due to the PSE report. The DIO or the Program Director alerted the resident upon receiving their PSE by email or verbally and provided feedback. This included appreciation for making the report and about corrective actions taken by the organization to address the issue raised because of their PSE. We also shared PSEs that resulted in specific hospital-wide improvements in patient safety with all the residents to foster transparency and encourage continuous reporting of patient safety events.

The last approach (November 2020 to March 2021) was aimed at improving the ability of residents to develop hypothetical solutions to patient safety events and to further increase resident engagement. We asked residents that submitted a PSE to consider what system-wide improvements would help to reduce the risk of the safety event they encountered. They were instructed to include potential risk reduction strategies or necessary process changes in their PSE report. Residents were encouraged to enter these solutions as a measurable safety goal in a specific, measurable, achievable, relevant, and time-bound (SMART) format. The associate program directors and chief residents received all PSEs submitted weekly with the reporter’s name redacted. A financial incentive of $10 was provided to the most well-formulated solution as determined by the associate program directors and chief residents.

Outcome measure

The outcome measure of interest was the number of PSEs entered by residents each month. The annual goal was 156 resident PSEs per academic year or 13 PSE per month (an average of four PSEs per resident). Monthly reports were provided to the Medical Executive Committee and the board of trustees. Data were aggregated monthly across the study period. At the end of each intervention, the number of PSE were calculated to determine changes by intervention. 

Statistical analysis

We recorded the number of PSE reported by residents monthly based on reports obtained from MorCare^TM^. Control charts were created and analyzed with QI Macros 2019 (Denver, CO: KnowWare International, Inc.). A c-chart tracked the number of PSE submitted by residents. Categorical variables were reported as percentages and associations were quantified using chi-squared and Fisher’s exact tests, as applicable. Fisher’s exact tests were used in cases where a combination of two variables resulted in any cell counts of five or less. A p-value equal to or less than 0.05 was considered to indicate a statistically significant relationship between two variables.

## Results

Over a 30-month period, residents submitted 314 PSEs. The mean number of PSEs submitted monthly increased from zero to two reports at baseline to 10.4 during the study period. The mean number of PSE increased to 5.8 (range: 2-11) at the end of the first intervention. Following the third intervention, the average number of reported PSE was 12.3 (range: 5-18) (Table 1).

**Table 1:** Patient safety event reporting by resident physicians by specific interventions over a 30-month period. PSE: patient safety event

Interventions	Mean PSE reports	Range
Baseline	0.6	0-2
Enhanced education	5.8	2-11
Engaging organizational leaders	8.8	2-13
Feedback process to the residents	12.3	5-18
Financial incentive	16.6	13-20

**Figure 1:**
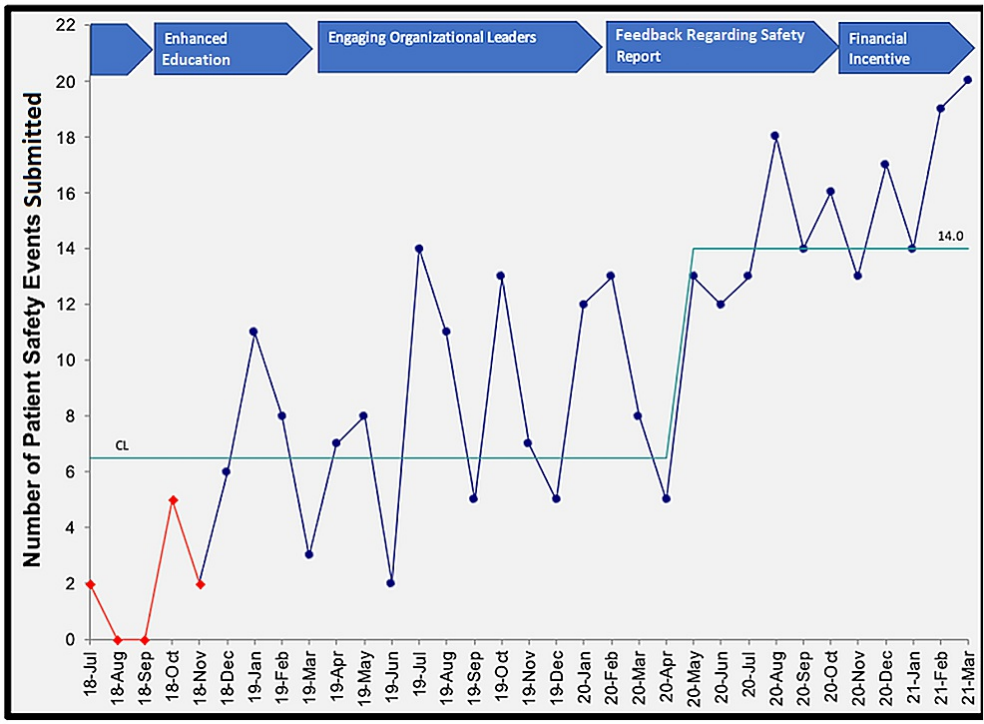
Run chart of patient safety events submitted by resident physicians. CL: centerline

We did not find a statistically significant difference between each intervention. However, there was a continued increase in the number of events reported across the study period, and this was sustained. The outcome of interest was not achieved after intervention one but was achieved in 27% and 62% of months following interventions two and three. By the end of the fourth intervention, our goal of >13 PSEs per month was consistently met. The most significant increase in reporting occurred when residents received positive timely feedback regarding their report and were notified regarding systematic changes made because of their report. The third test of change (feedback) had the least variation. This has been sustained six months following the completion of the study. We did not note a further increase in PSEs after offering financial incentives for the best hypothetical solutions to a PSE (Figure 1).

## Discussion

Our study describes the successful implementation of a longitudinal, multifaceted initiative to create a definitive and sustained increase in the number of PSEs reported by pediatric residents at our institution. The use of multiple strategies and the longitudinal nature of the project facilitated resident engagement and integration into the safety structure of our organization.

We achieved an increase in the number of PSE reports submitted by residents through various approaches; however, the most significant increase in PSE reports submission occurred during the third intervention, wherein the resident received acknowledgment of their submission and feedback regarding the outcomes of the PSE. It is well-known that healthcare personnel fail to obtain timely feedback about PSE reports [17-20]. In a study done by Farley et al., most hospitals surveyed that they do not have robust processes for sharing outcomes of adverse reports, and only 20-21% of hospitals thoroughly distribute and communicate action reports on identified safety events [21]. This leads to a lack of engagement in reporting safety issues and prevents efficient interactions among providers. Other studies in the literature describe ways to increase resident safety reporting in training programs [9-13]. Interventions and results are similar to our project; however, studies evaluating the impact of positive feedback are limited. To bridge this gap, we developed a process to ensure that resident PSEs were reviewed and acknowledged. The residents were notified of the strategies or risk reduction actions being taken to address the event they encountered. We found that this stimulated reporting among residents and resulted in an increase in PSE submissions. As residents will be future leaders in safety initiatives, they must understand their role as safety champions and their ability to affect organizational change. Training programs should prioritize establishing focused, individualized feedback mechanisms and enable residents to view the comments and actions that result following a PSE report to promote learning and facilitate sustained reporting. Furthermore, residents may have different perspectives and strategies on addressing a safety issue. Similar to our findings, Szymusiak et al. also demonstrated that residents felt that involvement in the post-PSE reporting process reinforced the value of making a report [21].

We found that strategies focused on enhanced educational interventions for residents, and education of institutional leaders about the need for resident involvement in our current systems resulted in modest changes in the number of PSE reports. Not all residents were able to attend the educational sessions due to scheduling conflicts, but we held repeat seminars monthly to ensure that the lack of improvement was not due to the incomplete training of all residents. Kaldjian et al. demonstrated that knowledge deficits were an essential factor in underreporting of PSE [14]. Similarly, previous studies investigating methods to improve PSE reporting have emphasized the importance of education [21-23]. The modest increase in our PSE rates with educational interventions alone supports the need for supplementary programs in addition to educational seminars when developing safety training programs for graduate medical education. Boike et al. showed that education and setting expectations for reporting as a stand-alone method to increase PSE reporting among residents might not be effective and insufficient in developing a meaningful long-term commitment [24].

Our last intervention encouraged residents to submit a PSE with hypothetical solutions to address the safety event they encountered. Interestingly, we found that residents faced difficulties developing specific improvement strategies to rectify and prevent a safety issue. This requires further exploration but may be due to a lack of familiarity with organizational structure and workflow and how to carry out an apparent cause analysis among resident physicians. To foster future involvement of residents in quality improvement and patient safety, it is crucial to examine residents’ understanding of the organizational policies and procedures for averting errors [25]. Interventions that incorporate information on how operational failures and human fallibilities are addressed at an organizational level may be valuable in promoting engagement and helping trainees see the bigger picture of patient safety [26,27]. Additionally, encouraging residents to participate in organizational quality and safety committees may help them to understand how PSEs are addressed at an institutional level.

Our study had several limitations. This project was conducted at a single institution with only one specialty represented and hence may not be reproducible to other specialties. Medical specialties historically report less than surgical specialties, and it is unknown whether our interventions would have a similar impact on surgical trainees [6]. Another limitation of our study is the lack of PSE submissions from the ambulatory settings, despite equivalent access to our online PSE reporting system. As a result, we do not have data to show the impact of our interventions in outpatient settings, and there are potential opportunities in the outpatient arenas that are not being captured. After the release of the Institute of Medicine's (IOM’s) report, Webster et al. demonstrated the inadequacies of patient safety monitoring in the ambulatory setting [28]. Lastly, we had very few PSE reported regarding nighttime safety events. As most safety events tend to happen around the time of handoffs, it would be interesting to look for ways to increase reporting at times of shift change and nighttime [29,30]. Despite these limitations, our study offers concrete strategies to increase PSE reporting among pediatric residents and integrate them into the safety culture of our institution. Further, developing a culture of reporting serious adverse incidents among pediatric residents is essential and we believe that by increasing PSE reporting, we will also be able to improve serious error reporting.

In the future, we plan to identify additional interventions to enhance reporting of safety issues by pediatric residents in the ambulatory setting. We also plan to include residents in organizational root cause analysis and similar processes specific to their reported patient safety events. These would provide opportunities for individual investment in building system-wide change and create prospects for future quality improvement projects. It is essential to recognize that trainees are often the first to witness and report safety events within the acute care setting and therefore integrating residents into the organizational safety culture is paramount [14-16]. Our interventions may be helpful to other institutions seeking to further encourage resident involvement in safety initiatives.

## Conclusions

This study describes the successful implementation of a multi-pronged, longitudinal approach to creating a definitive and sustained increase in the number of PSEs reported by pediatric residents at our institution. Interventions included enhanced educational interventions, cultivating recognition of the role of residents as reporters among institutional stakeholders, ensuring all residents receive meaningful feedback regarding their PSE, and providing a financial incentive to residents who were able to develop the best hypothetical solution to the PSE.

Our approach facilitated increased patient safety event reporting by pediatric residents, integration of residents into the organizational safety culture, and met accreditation requirements in a quantifiable way. As frontline providers, pediatric residents are well-positioned to identify and report adverse patient events; however, focused individualized feedback may be critical to facilitate a sustained response. Our strategies may be replicated at other residency programs seeking to establish resident involvement in safety initiatives. Further work is necessary to identify additional strategies to ensure residents gain an understanding of how patient safety events are addressed and prevented at an organizational level.
